# Entrapment of a spline from a multielectrode mapping catheter in the side hole of a nonsteerable guiding sheath, with dislodgement and subsequent suction retrieval of a proximal spline electrode

**DOI:** 10.1016/j.hrcr.2022.04.006

**Published:** 2022-04-09

**Authors:** Cassandra Voong, Eunice Lee, Daniel Sohinki

**Affiliations:** ∗ Department of Internal Medicine, Augusta University, Augusta, Georgia; † Medical College of Georgia School of Medicine, Augusta, Georgia; ‡ Division of Cardiac Electrophysiology, Augusta University, Augusta, Georgia

**Keywords:** Dislodgement, Electrode, Entrapment, Mapping catheter, Multielectrode, PentaRay, Spline

**Key Teaching Points**

• Use caution when maneuvering equipment that does not “feel right.”
• Examine all malfunctioning equipment once removed from the body to ensure all components have been successfully retrieved.
• The push towards “low-fluoro/no-fluoro” atrial fibrillation ablation should not impede the operator from direct examination of catheter and sheath behavior under fluoroscopy when unexpected equipment behavior or equipment malfunction is suspected.
• Best practices to avoid this complication include the avoidance of unoccupied sheaths in the heart when possible and argue for separate transseptal punctures when multiple left atrial sheaths are required.
• Multidisciplinary care approach may be warranted in managing complications.

## Introduction

Device entrapment during catheter ablation is a previously known complication. Although it is rare, the risk is generally felt to relate to coiled or looped elements on the device becoming entangled with cardiac structures (eg, the atrioventricular valves). Entrapment of catheters with linear elements (eg, the PentaRay catheter) is felt to be much less common, though this has been reported. We present a rare and interesting case of entrapment of a PentaRay catheter in the side hole of an SL1 sheath. A spline electrode was sheared off during attempts at catheter removal, and this was subsequently retrieved with manual suction through a steerable sheath.

## Case report

A 70-year-old man with a history of symptomatic paroxysmal atrial fibrillation (AF) and tachy-brady syndrome was referred to our institution for management of AF. Titration of medical therapy had been limited by symptomatic bradycardia; hence we chose to proceed with catheter ablation. He presented electively on the day of the procedure and was placed under general anesthesia, as is standard practice for AF ablation at our institution. On initial evaluation using intracardiac echocardiography, he was noted to have a severely aneurysmal interatrial septum. For that reason, we elected to perform a single transseptal puncture and to use the retained guidewire technique to guide a second sheath across the puncture site. Single transseptal puncture was performed using an SL1 sheath (8.5F) and BRK1 extra-sharp needle. After a long J-wire was advanced into the left upper pulmonary vein, a Vizigo steerable sheath (8.5F) (Biosense Webster Inc [part of Johnson & Johnson], Irvine, CA) was guided across the puncture site using a ThermoCool SmartTouch SF/SF FJ curve ablation catheter (Biosense Webster Inc [part of Johnson & Johnson], Irvine, CA). The PentaRay catheter (Biosense Webster Inc [part of Johnson & Johnson], Irvine, CA) was then advanced through the steerable sheath and was used to create a left atrial electroanatomic map. Of note, although we intended to place the PentaRay catheter back through the SL1 sheath after mapping was complete, no catheter was inserted through the SL1 sheath as a placeholder during mapping, and this was left vacant in the left atrium (LA).

**Figure 1:**
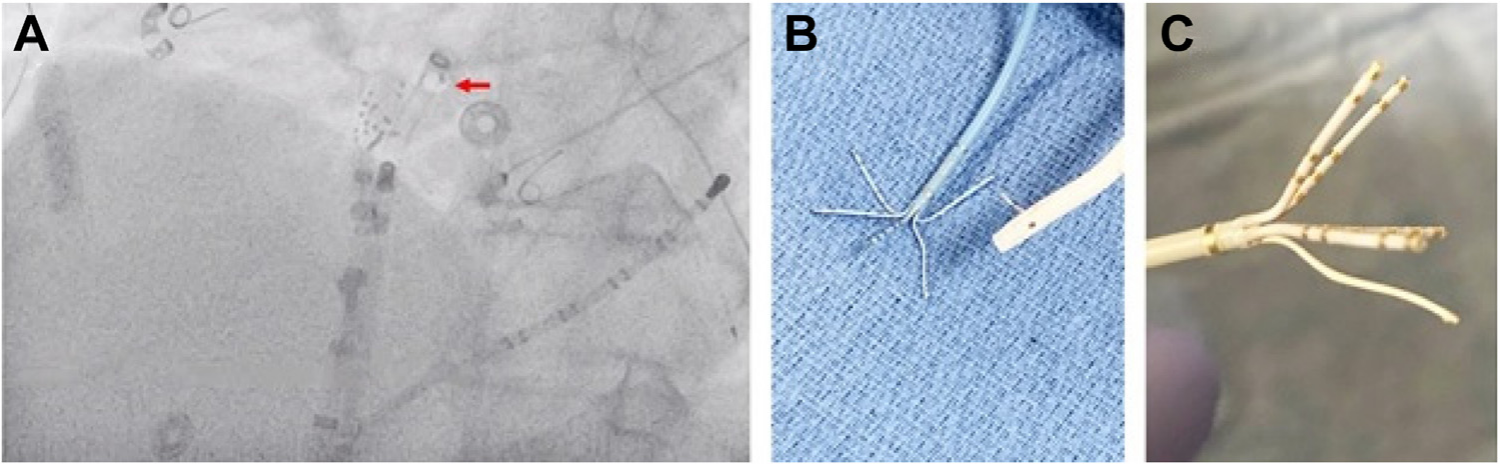
PentaRay spline entrapment. **A:** The spline that was sheared off during withdrawal into the SL1 sheath is visible on fluoroscopy in the left anterior oblique projection (*red arrow*). **B:** With the catheter and sheath removed from the body, the detached spline is visible retained in the SL1 sheath side hole. **C:** Close-up view of the PentaRay catheter with electrodes stripped off of the inferior-most spline.

During mapping, significant interaction was noted between the PentaRay and the tip of the SL1 sheath. The PentaRay was withdrawn into the steerable sheath and removed from the body. On inspection, it was noted that the electrodes had been stripped from 1 of the PentaRay splines, and on fluoroscopic examination the electrodes were noted to be retained in the side hole of the SL1 sheath (Figure 1).

**Figure 2:**
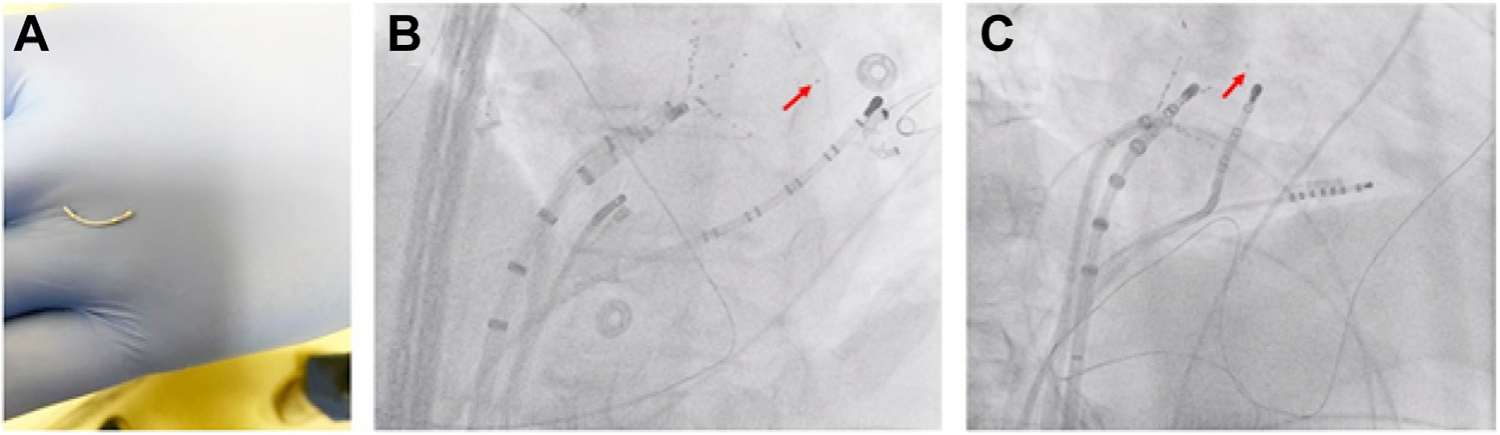
Dislodged electrode. **A:** With the spline fragment removed from the sheath, close examination revealed the proximal electrode to be missing. **B:** Left anterior oblique fluoroscopic projection with the retained electrode visible in the lateral left atrium (*red arrow*), presumed to be in the mitral apparatus, given its proximity to the tip of the coronary sinus catheter. **C:** Right anterior oblique projection, again with the retained electrode visualized in the anterior left atrium, confirming its position in or near the mitral apparatus.

The SL1 sheath was withdrawn to the level of the right iliac vein, and both the sheath and branch fragment were removed over a 0.035 inch Glidewire (Terumo, Shibuya City, Tokyo, Japan). On re-examination of the PentaRay branch fragment, it was noted that the proximal electrode from 1 of the splines was missing. Fluoroscopic examination revealed a retained electrode in the LA, likely entrapped in the mitral valve apparatus (Figure 2).

**Figure 3:**
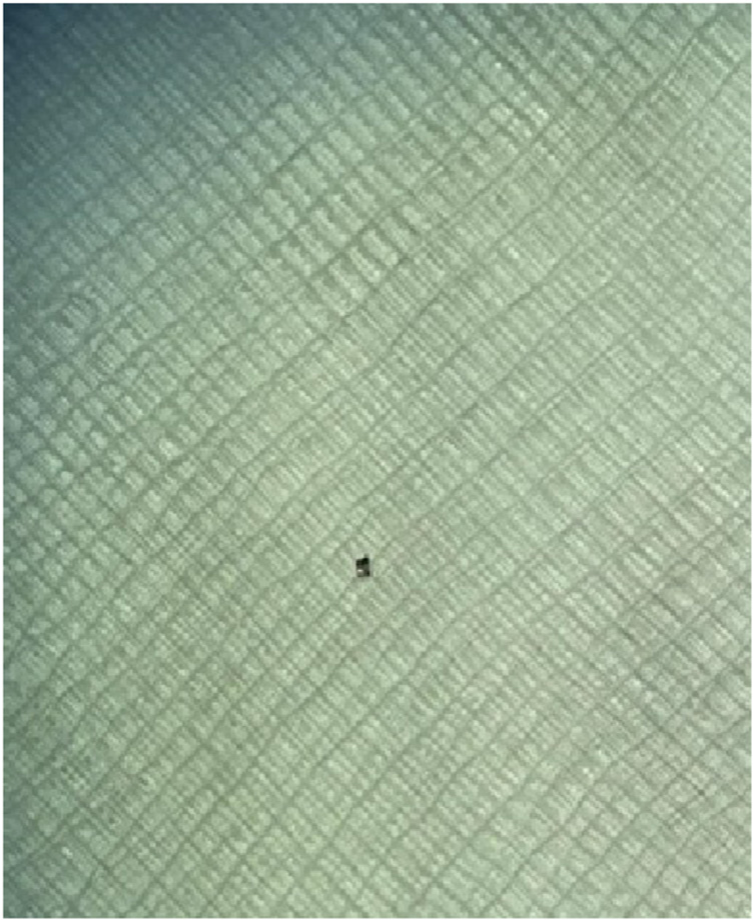
Retrieved spline electrode. As noted in the text, the Vizigo steerable sheath was advanced immediately adjacent to the electrode, confirmed by correlating fluoroscopic imaging, intracardiac echocardiography, and the electroanatomic map. A 60 cc syringe was then attached to the side port of the sheath, and manual suction was used to retrieve the electrode.

Consultation was then sought with interventional cardiology, interventional radiology, and cardiac surgery teams. Given the location of the electrode, it was felt that typical interventional techniques (eg, snare, vacuum removal) would be inadequate or infeasible to retrieve the electrode, and there were concerns regarding electrode embolization during preparation and performance of cardiac surgery. Intracardiac echocardiography was used to scan through the atrium, which located the electrode in the posterolateral mitral apparatus, and the CartoSound module was used to annotate its location on the electroanatomic map. Given its location, we decided to attempt fragment removal using suction through the steerable sheath. We did discuss the option of deploying a cerebral embolic protection device prior to attempted removal, though no staff were available with the required expertise to deploy the device, and we were not sure how much time we could wait to attempt removal of the fragment, given concerns about its stability in the mitral apparatus. We did also consider the possibility that the curve of the sheath might not be large enough to reach the electrode, but we felt that this approach might still be viable, given the availability of sheaths with multiple different curve sizes if needed. Using a combination of fluoroscopic guidance and visualization on the mapping system, the tip of the Vizigo sheath was advanced immediately adjacent to the electrode and suction from a 60 cc syringe attached to the side arm of the sheath was used to successfully remove the electrode (Figure 3). All equipment was replaced, and the ablation procedure was subsequently performed without complications.

## Discussion

There have been previous reports of device entrapment during catheter ablation of AF. Device entrapment can occur through interaction with other instruments used during the procedure^1^^,^^2^ or with the cardiovascular structures themselves. While rare, the most common concern is entrapment of circular mapping catheters (eg, the Lasso catheter [Biosense Webster]) in the mitral apparatus.^3^ Given the complexity of subvalvular structures and the nonlinear nature of the catheter, surgical intervention is often required for catheter retrieval.^4^ Catheter entanglement with other structures has been reported as well, including the vascular system involving the pulmonary veins^5^^,^^6^ and the ostium of the right internal iliac vein,^7^ mechanical mitral valves,^8^^,^^9^ or the Chiari network.^10^ In the presence of prosthetic heart valves, significant interaction and device embolization has been reported, even with straight, single-shaft catheters.^11^^,^^12^ The risk of entrapment of the PentaRay in the valvular apparatus is felt to be reduced, owing to the linear structure of its splines and its more pliable nature as compared to the Lasso. Although cases of PentaRay entrapment in a native mitral valve have been reported (Kastin, 2020, personal communication), overall prevalence of PentaRay entrapment requiring surgical removal is estimated at 0.0008% (Kastin, 2020, personal communication).

In this case, a PentaRay catheter was used. Its structure, which is composed of a unique star shape with 5 flexible soft branches and 22 1-mm-long electrodes distributed in a 4-4-4 mm arrangement,^13^ allows for atraumatic mapping in all cardiac chambers and theoretically poses less of a risk of device entanglement because of the pliable nature of the electrode splines. In addition, given its ability to provide multiple cardiac mapping data points simultaneously, a reduction of fluoroscopic time is needed, and fewer repositioning maneuvers of the catheter are required,^13^ which should also theoretically minimize the risk of entrapment. However, in our case the pliable, linear nature of the spline facilitated its entry into the side hole of the SL1 sheath. Once in the lumen, the spline was able to bend such that the shaft of the spline and side hole were no longer coaxial, which then allowed the proximal electrode to be caught in the hole. Traction applied to the shaft of the catheter outside of the body during attempted removal was enough to shear the electrodes off of the spline. Of note, there is 1 prior report where a branch of the PentaRay catheter was entrapped in the mitral valve and a distal portion of the branch was sheared off.^6^ However, the electrodes remained intact on that branch.

Several factors likely contributed to catheter entrapment in this case. First, a single transseptal puncture was used, given concern about inadvertent free-wall puncture with multiple transseptal attempts in the setting of an aneurysmal atrial septum. This necessarily creates contact between multiple transseptal sheaths and makes it more likely that equipment passing between both sheaths will interact. Second, no “placeholder” catheter was inserted through the SL1 sheath during mapping. Had a second catheter (eg, the ablation catheter) been used to occupy the lumen of the SL1 sheath, the PentaRay spline would have been much less likely to enter through the side hole far enough to become entrapped. Although such “placeholder” catheters are often used to prevent the sheath from scraping or puncturing through the endocardial surface of the LA, our case demonstrates an additional reason to occupy the lumen of the sheath—namely, prevention of interaction between equipment and/or cardiac structures with the sheath side holes.

Although the PentaRay catheter offers advantages with regard to intracardiac mapping, important elements of its design (eg, pliable, small-diameter splines) facilitated interaction with the SL1 sheath as noted above. Several different design considerations could potentially reduce the likelihood of this complication in the future, albeit with important tradeoffs. Altering the structural durability of the splines themselves would likely make them less likely to interact with other equipment or cardiac structures, though at the expense of less pliability and potentially greater risk of mechanical injury to the heart. Enlarging the tips of the individual splines would also be a consideration, though if this prevented the mapping electrodes from coming into direct contact with the endocardial surface, the accuracy of the anatomical and electrical data recorded from the catheter may be reduced. Ultimately, we feel that prevention of this complication through some of the considerations that we describe in this report is the best approach.

Our case also highlights the importance of examination of all malfunctioning equipment once removed from the body to ensure all components have been successfully retrieved. As is evident from Figure 2, the retained electrode would have been easily missed on simple fluoroscopic examination, especially given efforts to reduce fluoroscopic exposure during AF ablation. As noted above, the missing electrode was only discovered after direct examination of the damaged spline. This prompted scanning with fluoroscopy and subsequent discovery of the electrode in the mitral apparatus. Given the limitations of biplane fluoroscopy in direct localization of a structure in 3-dimensional space, we used the Vizigo sheath (which can be visualized on the mapping system) and the CartoSound module to identify both the sheath and electrode position in real time. This allowed us to ensure close proximity between the sheath tip and the electrode prior to attempted extraction without inadvertently mechanically dislodging the electrode with the tip of the sheath. Although the option was unavailable at the time of our case, deployment of a cerebral embolic protection device prior to attempted foreign body removal should be strongly considered, given the unknown risk of embolization using the approach we described.

Finally, our case serves as a cautionary tale regarding vigorous manipulation of equipment positioned inside the heart. Although the expected behavior of catheters and sheaths is learned through experience, it is best to use caution when maneuvering equipment that does not “feel right.” Though important, the push toward “low-fluoro/no-fluoro” AF ablation should not impede the operator from direct examination of catheter and sheath behavior under fluoroscopy when unexpected equipment behavior or equipment malfunction is suspected.

## Conclusion

This case illustrates a novel complication of catheter entrapment during intracardiac mapping using the PentaRay catheter in the presence of multiple long sheaths. Attempts at catheter removal resulted in shearing off of 1 of the spline electrodes, which was then able to be retrieved using manual suction through a steerable sheath. To our knowledge, this is the first report of PentaRay catheter entrapment in the side hole of a separate SL1 sheath. This case demonstrates best practices to avoid this complication, including avoidance of unoccupied sheaths in the heart when possible, and argues for separate transseptal punctures when multiple left atrial sheaths are required. Of note, the catheter was sent back to Johnson & Johnson for analysis of any structural abnormalities, though the results of this analysis are still pending at the time of this writing. Additionally, our case demonstrates the importance of multidisciplinary care in managing complications and provides a viable solution if operators encounter this complication in the future.
